# Ca^**2+**^ Signaling in Cytoskeletal Reorganization, Cell Migration, and Cancer Metastasis

**DOI:** 10.1155/2015/409245

**Published:** 2015-04-22

**Authors:** Feng-Chiao Tsai, Guan-Hung Kuo, Shu-Wei Chang, Pei-Ju Tsai

**Affiliations:** ^1^Department of Pharmacology, National Taiwan University College of Medicine, No. 1, Section 1, Ren-Ai Road, Taipei 100, Taiwan; ^2^Department of Internal Medicine, National Taiwan University Hospital, No. 7 Chung-Shan South Road, Taipei 100, Taiwan

## Abstract

Proper control of Ca^2+^ signaling is mandatory for effective cell migration, which is critical for embryonic development, wound healing, and cancer metastasis. However, how Ca^2+^ coordinates structural components and signaling molecules for proper cell motility had remained elusive. With the advance of fluorescent live-cell Ca^2+^ imaging in recent years, we gradually understand how Ca^2+^ is regulated spatially and temporally in migrating cells, driving polarization, protrusion, retraction, and adhesion at the right place and right time. Here we give an overview about how cells create local Ca^2+^ pulses near the leading edge, maintain cytosolic Ca^2+^ gradient from back to front, and restore Ca^2+^ depletion for persistent cell motility. Differential roles of Ca^2+^ in regulating various effectors and the interaction of roles of Ca^2+^ signaling with other pathways during migration are also discussed. Such information might suggest a new direction to control cancer metastasis by manipulating Ca^2+^ and its associating signaling molecules in a judicious manner.

## 1. Introduction

Calcium is one of the most important chemical elements for human beings. At the organismic level, calcium together with other materials composes bone to support our bodies [1]. At the tissue level, the compartmentalization of calcium ions (Ca^2+^) regulates membrane potentials for proper neuronal [2] and cardiac [3] activities. At the cellular level, increases in Ca^2+^ trigger a wide variety of physiological processes, including proliferation, death, and migration [4]. Aberrant Ca^2+^ signaling is therefore not surprising to induce a broad spectrum of diseases in metabolism [1], neuron degeneration [5], immunity [6], and malignancy [7]. However, though tremendous efforts have been exerted, we still do not fully understand how this tiny divalent cation controls our lives.

Such a puzzling situation also exists when we consider Ca^2+^ signaling in cell migration. As an essential cellular process, cell migration is critical for proper physiological activities, such as embryonic development [8], angiogenesis [9], and immune response [10], and pathological conditions, including immunodeficiency [11], wound healing [12], and cancer metastasis [13]. In either situation, coordination between multiple structural (such as F-actin and focal adhesion) and regulatory (such as Rac1 and Cdc42) components is required for cell migration processes (or modules), including polarization, protrusion, retraction, and adhesion [8]. Since Ca^2+^ signaling is meticulously controlled temporally and spatially in both local and global manners, it serves as a perfect candidate to regulate cell migration modules. However, although the significant contribution of Ca^2+^ to cell motility has been well recognized [14], it had remained elusive how Ca^2+^ was linked to the machinery of cell migration. The advances of live-cell fluorescent imaging for Ca^2+^ and cell migration in recent years gradually unravel the mystery, but there is still a long way to go.

In the present paper, we will give a brief overview about how Ca^2+^ signaling is polarized and regulated in migrating cells, its local actions on the cytoskeleton, and its global effect on cell migration and cancer metastasis. The strategies employing Ca^2+^ signaling to control cell migration and cancer metastasis will also be discussed.

## 2. History: The Journey to Visualize Ca^2+^ in Live Moving Cells

The attempt to unravel the roles of Ca^2+^ in cell migration can be traced back to the late 20th century, when fluorescent probes were invented [15] to monitor intracellular Ca^2+^ in live cells [16]. Using migrating eosinophils loaded with Ca^2+^ sensor Fura-2, Brundage et al. revealed that the cytosolic Ca^2+^ level was lower in the front than the back of the migrating cells. Furthermore, the decrease of regional Ca^2+^ levels could be used as a marker to predict the cell front before the eosinophil moved [17]. Such a Ca^2+^ gradient in migrating cells was also confirmed by other research groups [18], though its physiological significance had not been totally understood.

In the meantime, the importance of local Ca^2+^ signals in migrating cells was also noticed. The use of small molecule inhibitors and Ca^2+^ channel activators suggested that local Ca^2+^ in the back of migrating cells regulated retraction and adhesion [19]. Similar approaches were also recruited to indirectly demonstrate the Ca^2+^ influx in the cell front as the polarity determinant of migrating macrophages [14]. Unfortunately, direct visualization of local Ca^2+^ signals was not available in those reports due to the limited capabilities of imaging and Ca^2+^ indicators in early days.

The above problems were gradually resolved in recent years with the advance of technology. First, the utilization of high-sensitive camera for live-cell imaging [20] reduced the power requirement for the light source, which eliminated phototoxicity and improved cell health. A camera with high sensitivity also improved the detection of weak fluorescent signals, which is essential to identify Ca^2+^ pulses of nanomolar scales [21]. In addition to the camera, the emergence of genetic-encoded Ca^2+^ indicators (GECIs) [22, 23], which are fluorescent proteins engineered to show differential signals based on their Ca^2+^-binding statuses, revolutionized Ca^2+^ imaging. Compared to small molecule Ca^2+^ indicators, GECIs' high molecular weights make them less diffusible, enabling the capture of transient local signals. Furthermore, signal peptides could be attached to GECIs so the recombinant proteins could be located to different compartments, facilitating Ca^2+^ measurements in different organelles. Such tools dramatically improved our knowledge regarding the dynamic and compartmentalized characteristics of Ca^2+^ signaling.

With the above techniques, “Ca^2+^ flickers” were observed in the front of migrating cells [18], and their roles in cell motility were directly investigated [24]. Moreover, with the integration of multidisciplinary approaches including fluorescent microscopy, systems biology, and bioinformatics, the spatial role of Ca^2+^, including the Ca^2+^ gradient in migrating cells, was also gradually clarified [25]. Our present understanding about Ca^2+^ signaling in migrating cells is briefly summarized as follows.

## 3. Ca^2+^ Transporters Regulating Cell Migration

### 3.1. Generators of Local Ca^2+^ Pulses: Inositol Triphosphate (IP_3_) Receptors and Transient Receptor Potential (TRP) Channels (Figure 1)

For a polarized cell to move efficiently, its front has to coordinate activities of protrusion, retraction, and adhesion [8]. The forward movement starts with protrusion, which requires actin polymerization in lamellipodia and filopodia, the foremost structure of a migrating cell [8, 13, 26]. At the end of protrusion, the cell front slightly retracts and adheres [27] to the extracellular matrix. Those actions occur in lamella, the structure located behind lamellipodia. Lamella recruits myosin to contract and dissemble F-actin in a treadmill-like manner and to form nascent focal adhesion complexes in a dynamic manner [28]. After a successful adhesion, another cycle of protrusion begins with actin polymerization from the newly established cell-matrix adhesion complexes. Such protrusion-slight retraction-adhesion cycles are repeated so the cell front would move in a caterpillar-like manner.

For the above actions to proceed and persist, the structural components, actin and myosin, are regulated in a cyclic manner. For actin regulation, activities of small GTPases, Rac, RhoA, and Cdc42 [29], and protein kinase A [30] are oscillatory in the cell front for efficient protrusion. For myosin regulation, small local Ca^2+^ signals are also pulsatile in the junction of lamellipodia and lamella [24]. Those pulse signals regulate the activities of myosin light chain kinase (MLCK) and myosin II, which are responsible for efficient retraction and adhesion [31, 32]. Importantly, due to the extremely high affinity between Ca^2+^-calmodulin complexes and MLCK [33], small local Ca^2+^ pulses in nanomolar scales are sufficient to trigger significant myosin activities.

The critical roles of local Ca^2+^ pulses in migrating cells raise the question where those Ca^2+^ signals come from. In a classical signaling model, most intracellular Ca^2+^ signals originate from endoplasmic reticulum (ER) through inositol triphosphate (IP_3_) receptors [34, 35], which are activated by IP_3_ generated via receptor-tyrosine kinase- (RTK-) phospholipase C (PLC) signaling cascades. It is therefore reasonable to assume that local Ca^2+^ pulses are also generated from internal Ca^2+^ storage, that is, the ER. In an in vitro experiment, when Ca^2+^ chelator EGTA was added to the extracellular space, local Ca^2+^ pulses were not immediately eliminated from the migrating cells [24], supporting the above hypothesis. Moreover, pan-RTK inhibitors that quenched the activities of RTK-PLC-IP_3_ signaling cascades reduced local Ca^2+^ pulses efficiently in moving cells [25]. The observation of enriched RTK and PLC activities at the leading edge of migrating cells was also compatible with the accumulation of local Ca^2+^ pulses in the cell front [25]. Therefore, polarized RTK-PLC-IP_3_ signaling enhances the ER in the cell front to release local Ca^2+^ pulses, which are responsible for cyclic moving activities in the cell front.

In addition to RTK, the readers may wonder about the potential roles of G protein-coupled receptors (GPCRs) on local Ca^2+^ pulses during cell migration. As the major pathway to activate PLC, GPCRs coupled to the G_*α*q/11_ subunit [36] trigger the cleavage of phosphatidylinositol (4,5)-bisphosphate (PIP_2_) by PLC to generate diacylglycerol (DAG) and IP_3_, which subsequently releases Ca^2+^ from the ER as Ca^2+^ pulses and spikes [37]. Indeed, Ca^2+^ oscillations induced by GPCR pathways have been observed in various cell types [38, 39]. However, the GPCRs coupled to G_*α*q/11_ and PLC include serotonergic, adrenergic, muscarinic, glutamatergic, and histamine receptors [37], most of which do not directly affect cell migration. In contrast, growth factors contributing to cell migration, such as fibroblast growth factor (FGF) [40], epidermal growth factor (EGF) [41], and vascular endothelial growth factor (VEGF) [42], activate RTK signaling pathways. Therefore, it is more likely for the RTK rather than GPCR pathway to be responsible for local Ca^2+^ pulses in migrating cells. Nonetheless, more studies are required to clarify this important question.

The readers may also be curious how nonpulsatile RTK-PLC signaling generates oscillatory local Ca^2+^ pulses. In fact, IP_3_-induced Ca^2+^ oscillation has been reported repeatedly in various physiological circumstances [34, 38, 43, 44]. One possibility is that RTK signaling sensitizes IP_3_ receptors in the front ER but does not directly open those Ca^2+^ channels. Alternatively, the cyclic Ca^2+^ channel opening could be triggered by Ca^2+^-induced Ca^2+^ release (CICR), which is the activation of IP_3_ receptors by small changes of local Ca^2+^ levels [45, 46]. In the protruding cell front, the change of membrane tension may open stretch-activated transient receptor potential (TRP) channels [47], offering the required CICR. Indeed, TRP channels have been extensively reported as major contributors for cell migration and cancer metastasis [48]. Specifically, TRPM7 has been revealed to enhance cancer cell metastasis [49, 50], by mediating Ca^2+^ influx [51], altering Ca^2+^ flickers [18, 52], and regulating cell-matrix adhesion [53]. Therefore, oscillatory small Ca^2+^ pulses in the migrating cell front are probably the integrated results of polarized RTK signaling interacting with pulsatile membrane stretch and TRP channel opening, to release Ca^2+^ periodically from the front ER.

### 3.2. Maintainers of Basal Ca^2+^ Levels and Gradient: Sarcoplasmic/Endoplasmic Reticulum Ca^2+^-ATPase (SERCA) and Plasma Membrane Ca^2+^-ATPase (PMCA) (Figure 2)

The fact that tiny cyclic Ca^2+^ signals induce significant changes of cell motility implies that the basal cytosolic Ca^2+^ level, especially that at the front of migrating cells, has to be extremely low, so the cell migration machinery, specifically myosin and focal adhesion complexes, can promptly respond to small Ca^2+^ changes. To achieve the above goal, the migrating cells meticulously utilize two types of Ca^2+^-ATPase pumps, sarcoplasmic/endoplasmic reticulum Ca^2+^-ATPase (SERCA) and plasma membrane Ca^2+^-ATPase (PMCA).

#### 3.2.1. SERCA Pumps Cytosolic Ca^2+^ into the ER

SERCA is transmembranously located at the ER, continuously pumping cytosolic Ca^2+^ into the ER lumen with fast speed and high affinity [4]. Although their activities are slightly regulated by phospholamban and protein kinase A (PKA) [54], these pumps maintain the internal Ca^2+^ storage with high fidelity. Once SERCA is inactivated, the ER luminal Ca^2+^ leaks out to the cytoplasm in no time [55]. The resulting high cytosolic Ca^2+^ will saturate MLCK and induce persistent contraction of myosin [25], rendering front protrusion not possible. Furthermore, SERCA dysfunction dramatically reduces the ER luminal Ca^2+^, disabling further Ca^2+^ signaling through IP_3_ receptors. Hence, SERCA is essential for physiological and pathological cell migration. It is therefore not surprising to see aberrant SERCA expressions in cancer progression, invasion, and metastasis [56, 57].

#### 3.2.2. SERCA Is Not Responsible for the Cytosolic Ca^2+^ Gradient in Migrating Cells

Since SERCA continuously and efficiently removes Ca^2+^ out of the cytoplasm into the ER, it is convenient to hypothesize that cytosolic Ca^2+^ gradient in migrating cells results from differential SERCA activities. If the SERCA activity was higher in the front than in the back, more Ca^2+^ in the front cytosol would be pumped into the front ER, resulting in the low-in-front, high-in-back Ca^2+^ gradient in the cytoplasm and a reverse (high-in-front, low-in-back) gradient in the ER. However, blocking SERCA activities with small molecule inhibitors caused a paradoxical increase of Ca^2+^ gradient, in addition to the global increase of cytosolic Ca^2+^ [25]. Monitoring intra-ER Ca^2+^ with the T1ER FRET probe [58] also revealed a low-in-front, high-in-back Ca^2+^ gradient when the cell moved [25]. Therefore, though a pivotal molecule keeps the cytosol Ca^2+^ free at the basal status, SERCA does not contribute to the Ca^2+^ gradient in migrating cells.

#### 3.2.3. Differential PMCA Activities Keep [Ca^2+^] in the Front Lower Than the Back during Cell Migration

Based on the above data, Ca^2+^ pumps at the plasma membrane might be better candidates for the Ca^2+^ gradient during cell migration. Similar to SERCA, PMCA also continuously removes cytosolic Ca^2+^, by pumping it to the extracellular space [4, 59]. Unlike SERCA, recent evidence revealed that PMCA inhibitors and siRNA reduced Ca^2+^ gradient and cell motility during cell migration [25]. Direct measurement of Ca^2+^ efflux through plasma membrane also demonstrated an enhancement of PMCA activity by 30–50% in the front of migrating cells [25]. Hence, differential PMCA activities might account for the Ca^2+^ gradient during cell migration.

It is still not totally understood how cells adjust local PMCA activities to make them high in the front and low in the back. Several modulators have been demonstrated to regulate PMCA, including calmodulin [60], PKA [61], and calpain [62]. Whether those proteins could be spatially regulated inside the cells remains elusive. In addition, PMCA was enriched in the front plasmalemma of moving cells [25], suggesting that its differential distribution might account for the well-recognized front-low, back-high Ca^2+^ gradient during cell migration. Still, how PMCA is accumulated in the cell front requires further investigation.

### 3.3. Maintainers of Ca^2+^ Homeostasis during Migration: Store-Operated Ca^2+^ (SOC) Influx (Figure 3)

SOC influx is an essential process to maintain internal Ca^2+^ storage [63] for IP_3_ receptor-based Ca^2+^ signaling, during which the luminal ER Ca^2+^ is evacuated. After IP_3_-induced Ca^2+^ release, although Ca^2+^ can be recycled back to the ER through SERCA, a significant amount of cytosolic Ca^2+^ will be pumped out of the cell through PMCA, resulting in the depletion of internal Ca^2+^ storage. To rescue this, low luminal Ca^2+^ activates STIM1 [55, 64], which is a membranous protein located at the ER and transported to the cell periphery by microtubules [65, 66]. Active STIM1 will be translocated to the ER-plasma membrane junction [67], opening the Ca^2+^ influx channel ORAI1 [68, 69]. Ca^2+^ homeostasis could therefore be maintained during active signaling processes including cell migration.

Since the identification of STIM1 and ORAI1 as the major players of SOC influx, numerous reports have emerged confirming their significant roles in cell migration and cancer metastasis (Tables 1 and 2). Although it is reasonable for those Ca^2+^-regulatory molecules to affect cell migration, the molecular mechanism is still not totally clear. Recent experimental evidence implied that STIM1 helped the turnover of cell-matrix adhesion complexes [7, 25], so SOC influx may assist cell migration by maintaining local Ca^2+^ pulses in the front of migrating cells. In a moving cell, local Ca^2+^ pulses near its leading edge result in the depletion of Ca^2+^ in its front ER. Such depletion subsequently activates STIM1 at the cell front. Compatible with the above assumption, more STIM1 was translocated to the ER-plasma membrane junction in the cell front compared to its back during cell migration [25]. Moreover, in addition to the ER and plasma membrane, STIM1 is also colocalized with EB1 [65, 66], the cargo protein located at the plus ends of microtubules. Further experiments revealed that STIM1 was actively transported to the front ER assisting cell migration [25]. Therefore, STIM1 together with other Ca^2+^ channels is meticulously regulated in a spatial manner maintaining cell polarity and motility.

## 4. Ca^2+^ Effectors for Cell Migration (Figure 4)

As described above, intracellular Ca^2+^ is regulated locally and globally for effective cytoskeletal remodeling, cell migration, and cancer metastasis. Ca^2+^ pulses and spikes occur at the right place and right time, activating numerous downstream structural and signaling targets, which have been investigated separately over the past decades. The clarification of Ca^2+^ signaling in recent years has dramatically improved our understanding about how those components are regulated temporally and spatially in migrating cells. However, such advancement has revealed more questions than answers. More efforts are required to resolve those problems in the future.

### 4.1. Signaling-Related Targets

#### 4.1.1. Protein Kinase C (PKC)

PKC is a typical downstream target of Ca^2+^ in receptor tyrosine kinase signaling pathways, during which the growth factor binds to the receptor and activates its tyrosine kinase through dimerization and autophosphorylation [70]. The resulting activation of PLC generates diacylglycerol (DAG) and IP_3_, which subsequently induces Ca^2+^ release from the ER. DAG and Ca^2+^ then bind separately to the C1 and C2 domains of classical PKC (PKC*α*, *β*, and *γ*) [71]. Depending on the substrate, classical PKC regulates a wide variety of physiological processes, including cell migration [72]. The action could be direct via phosphorylation or indirect through transcriptional activation.

The classical PKC family has direct and significant impact on cell migration. PKC*α* is enriched in the front of migrating cells [14]. It directly phosphorylates Rho GTPases and multiple components of focal adhesion complexes, regulating the remodeling of cell-matrix adhesion (see [73] for a more comprehensive review). PKC*β* phosphorylates the heavy chains of myosin II, inhibiting myosin contraction and facilitating the process of directional determination in migrating cells [74–77]. How these PKCs respond to spatiotemporal Ca^2+^ signaling and coordinate for effective moving activities requires further investigation.

Besides classical PKCs, atypical PKCs [70] also regulate the polarity of migrating cells. Unlike classical PKCs, those PKCs do not require DAG or Ca^2+^ for activation [70]. Together with Rho GTPases [78, 79], these PKCs might be actively involved in the dynamic processes of cell protrusion and adhesion [78, 80]. How these actions synchronize with the Ca^2+^ dynamics during cell migration also awaits more research in the future.

#### 4.1.2. Rho GTPases

Rho GTPases, including Rac1, RhoA, and Cdc42, have been known as the key components for the regulation of actin dynamics [81]. It is therefore not surprising to see their active involvement in cell migration. Spatially, in a simplified model, these GTPases are enriched at specific structures of a migrating cell, Rac1 in lamellipodia, RhoA around focal adhesion complexes, and Cdc42 near filopodia [8]. Temporally, activities of these GTPases are pulsatile and also synchronized to the cyclic lamellipodial activities in the front of migrating cells [29]. Therefore, Rho GTPases, similar to Ca^2+^ [24], exert actions at the right place and right time for proper actin remodeling and efficient cell migration.

Although the present data reveals no evidence of direct binding between Ca^2+^ and Rho GTPases, it is reasonable to expect their mutual interactions considering their perfect coordination during cell migration [24, 29, 30]. Such speculation is supported by the observation that blocking Ca^2+^ influx at the leading edges of polarized macrophages resulted in the disassembly of actin filaments and lamellipodia activities [14]. The facts that constitutively active Rac1 fully rescued the effects of SOC influx inhibition in migrating breast cancer cells [82] also indicate the regulatory role of Ca^2+^ on Rho GTPases. Moreover, the transamidation of Rac1 was shown to be dependent on intracellular Ca^2+^ and calmodulin in rat cortical cells, suggesting the biochemical link between Rho GTPases and Ca^2+^ signaling [83]. Hopefully more studies will be conducted in the near future to clarify the mechanism of how Ca^2+^ interacts with Rho GTPases.

### 4.2. Cytoskeleton-Related Targets

#### 4.2.1. Myosin II

As mentioned above, local Ca^2+^ pulses at the junction of lamellipodia and lamella activate MLCK [24], which subsequently phosphorylates myosin light chain and triggers myosin contraction. It is worth noticing that the affinity between MLCK and myosin-calmodulin is extremely high, with the dissociation constant (*K*_*d*_) of about 1 nM [33]. Therefore, a slight increase of local Ca^2+^ concentration is sufficient to induce significant activation of MLCK and subsequent contraction of myosin II. Moreover, the high sensitivity of MLCK to Ca^2+^ implies that the front cytoplasm has to be free of Ca^2+^ at the basal status, so MLCK can be inactive at baseline but respond to small rises of Ca^2+^ promptly. Such design justifies the physiological importance of the front-low, back-high Ca^2+^ gradient in migrating cells.

In cell migration, the immediate effect of myosin contraction is the retraction of actin bundles, which not only facilitates the disassembly of F-actin at lamella but also allows the protruding front to attach to the extracellular matrix [28, 31]. In addition, myosin contraction also stabilizes nascent focal adhesion complexes in the front of migrating cells [32, 84]. This is probably because these contractions apply traction force on the complexes through actin bundles binding to them. Such force subsequently induces remodeling and stabilization of the components in focal adhesion. Therefore, through MLCK and myosin II, local Ca^2+^ pulses are tightly linked to the oscillatory dynamics of cell protrusion, retraction, and adhesion.

#### 4.2.2. Actin

Besides myosin, Ca^2+^ also affects the dynamics of actin, the major component of cytoskeleton [85, 86]. Although Ca^2+^ does not directly bind to actin, it affects the activities of multiple actin regulators. First of all, Ca^2+^ activates protein kinase C and calmodulin-dependent kinases, both of which interact with actin affecting its dynamics [87–89]. Secondly, as also described above, Ca^2+^ signaling regulates the Rho GTPases [14], which are mandatory for the formation of actin bundles for lamellipodia, focal adhesion complexes, and filopodia [8], the major components for cell migration. In addition, the F-actin severing protein cofilin [90, 91] also depends on the cytosolic Ca^2+^ for its proper activity. Moreover, myosin, as one the major actin regulators, is totally dependent on Ca^2+^ for its proper activity [24]. Therefore, though not a direct regulator, Ca^2+^ modulates actin dynamics through multiple signaling pathways and structural molecules.

### 4.3. Adhesion-Related Targets

#### 4.3.1. Calpain

In addition to kinase activities and physical force, Ca^2+^ also affects cell migration through protein cleavage and degradation. Calpain, as a Ca^2+^-dependent intracellular protease [92, 93], is involved in the turnover of stable focal adhesion complexes, probably at the rear end of migrating cells. Calpain has been revealed to cleave several components of the focal adhesion complex, including talin [94], paxillin [95], and focal adhesion kinases [96], compatible with previous reports showing that Ca^2+^ influx at the back of migrating cells facilitated retraction and detachment at their rear ends [97]. Beside focal adhesion, calpain also degrades PMCA [62]. Since there is an inverse correlation between the front-back gradients of Ca^2+^ and PMCA in migrating cells [25], decreased amount of PMCA in the cell back may result from the higher Ca^2+^ level and higher calpain activity in the back than in the front. However, such speculation requires more experimental data to be validated.

#### 4.3.2. Pyk2 and Other Molecules

In addition to calpain, several adhesion-related proteins are also regulated by Ca^2+^, including Pyk2, plectin, and matrix metallopeptidases.

As a cytoplasmic protein tyrosine kinase, Pyk2 is activated by intracellular Ca^2+^ and protein kinase C [98]. It regulates the activities of focal adhesion kinase and GRB2, affecting focal adhesion complexes [99] and the MAP kinase signaling pathway [98]. In human cervical cancer cells, aberrant SOC influx changes focal adhesion dynamics through Pyk2 dysregulation [7].

Ca^2+^ also regulates the conveyance of integrin-based signaling into the cytoskeleton, with its interaction with plectin, the bridge between integrin complexes and actin filaments. Recent biochemical and biophysical evidence indicated that the binding of plectin 1a with Ca^2+^ effectively decreased its interactions with integrin *β* and with F-actin, decoupling cell-matrix adhesion with cytoskeletal structures [100, 101]. We may speculate that, with proper temporal and spatial Ca^2+^ regulation, cells could determine how many environmental signals would be conducted into the cells for cytoskeleton modification. More studies are required to clarify the above hypothesis.

Furthermore, matrix metallopeptidases (MMP), as facilitating factors for cancer metastasis, are also regulated by intracellular Ca^2+^. In prostate cancer, increased expression of TRPV2 elevated cytosolic Ca^2+^ levels, which enhanced MMP9 expression and cancer cell aggressiveness [102]. Further investigation in melanoma cells revealed that increased intracellular Ca^2+^ induced the binding of Ca^2+^-modulating cyclophilin ligand to basigin, stimulating the production of MMP [103]. Therefore, Ca^2+^ not only modulates the outside-in (integrin to actin) signaling but also regulates the inside-out (Ca^2+^ to MMP) signaling for cell migration and cancer metastasis.

## 5. Future: Interactions between Ca^2+^ and Other Signaling Pathways

Regarding the complicated temporal and spatial regulation of Ca^2+^ signaling in migrating cells, we would expect extensive interactions between Ca^2+^ and other signaling modules during cell migration. Indeed, though still preliminary, recent work has revealed potential cross talk between Ca^2+^ and other pathways controlling cell motility. These findings will shed new light on our pilgrimage toward a panoramic view of cell migration machinery.

### 5.1. Interactions between SOC Influx and Cell-Matrix Adhesion

In the present model, SOC influx maintains Ca^2+^ storage in the ER, which releases local Ca^2+^ pulses to enhance the formation of nascent focal adhesion complexes [25]. Therefore, the inhibition of SOC influx should weaken cell-matrix adhesion. Interestingly, STIM1, the Ca^2+^ sensor for the activation of the SOC influx, had been reported as an oncogene [82] or a tumor suppressor gene [104] by different groups. Furthermore, although most recent research suggested a positive role of STIM1 on cancer cell motility (Table 1), other reports revealed the opposite results in primary cells (Table 2). Therefore, effects of SOC influx on cell migration might vary under different circumstances.

One possible explanation of the confusing results uses the interaction between Ca^2+^ and basal cell-matrix adhesion. Primary cells are usually well attached to the matrix, so further enhancing their adhesion capability might trap them in the matrix and deter them from moving forward. In contrast, metastatic cancer cells often have weak cell-matrix adhesion, so strengthening their attachment to the matrix facilitates the completion of cell migration cycles. Indeed, recent evidence suggested that, in an in vitro cell migration assay [25], SOC influx might increase or decrease the motility of the same cell type depending on concentrations of fibronectin for the cells to attach. Though further explorations are required to validate the present data, the combination of SOC influx inhibition and cell-matrix adhesion blockage might be a novel approach to prevent cancer metastasis.

### 5.2. Coordination between the Oscillations of Ca^2+^ and Rho GTPases

Previous reports have revealed the oscillatory activities of Rho GTPases in the front of migrating cells, including Rac1, RhoA, and Cdc42 [29, 30]. These molecules regulate actin dynamics and coordinate with the pulsatile lamellipodial activities. Since the oscillation of local Ca^2+^ pulses synchronize with the retraction phases of lamellipodial cycles [24], there probably exists cross talk between Ca^2+^ signaling and Rho GTPases. Clarifying how these molecules are regulated to coordinate with each other will dramatically improve our understanding of lamellipodia and help developing better strategies to control physiological and pathological cell migration.

### 5.3. Link between Ca^2+^, RTK, and Lipid Signaling

The meticulous spatial control of Ca^2+^ signaling in migrating cells, together with the enrichment of RTK, phosphatidylinositol (3,4,5)-triphosphate (PIP_3_), and DAG in the cell front [25], reveals the complicated nature of the migration polarity machinery. How these signaling pathways act together to determine the direction for cells to move remains elusive and requires more research. In addition, understanding how nonpulsatile RTK and lipid signaling exert effects on oscillatory Ca^2+^ pulses will improve our knowledge about the spatial and temporal regulation of signal transduction inside the cells. Such information will further enhance our capability to develop novel strategies targeting pathological processes and manipulating diseases.

## Figures and Tables

**Figure 1:**
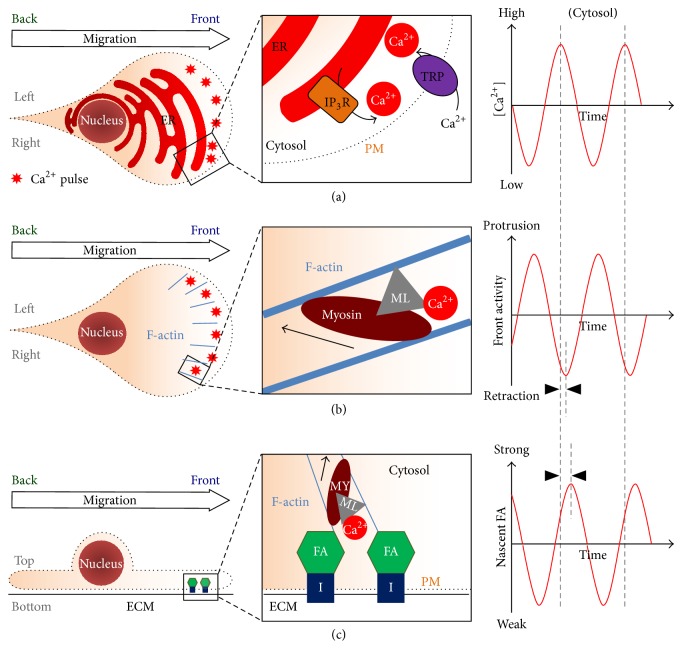
Local Ca^2+^ pulses control retraction and adhesion around the leading edge of migrating cells. (a) Polarized receptor tyrosine kinase (RTK) signaling generates inositol triphosphate (IP_3_) in front of migrating cells, which sensitizes IP_3_ receptors (IP_3_R) to release Ca^2+^ periodically from the endoplasmic reticulum (ER). IP_3_R are also triggered by Ca^2+^-induced Ca^2+^ release (CICR), which originates from transient receptor potential (TRP) channels, mainly TRPM7. (b) Local Ca^2+^ pulses activate myosin light chain kinase (MLCK, shown as ML in the illustration), which phosphorylates myosin II for proper actin treadmilling and recycling. (c) Local Ca^2+^ pulse-triggered myosin contraction also enhances the formation of focal adhesion (FA) complexes, probably via force-induced positive feedback. Please notice the temporal correlation (as shown by dotted lines and arrowheads) and oscillatory dynamics between local Ca^2+^ pulses, front retraction, and FA. MY: myosin II; ML: myosin light chain kinase; I: integrin; ECM: extracellular matrix.

**Figure 2:**
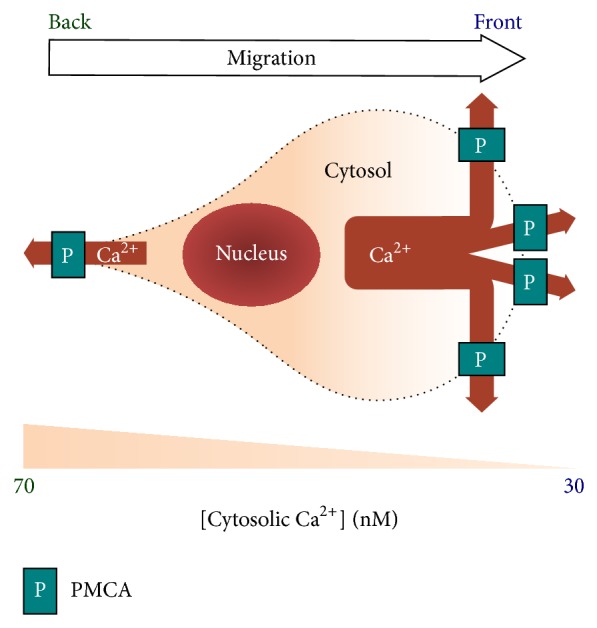
Cytosolic Ca^2+^ levels are low in the front and high in the back of the migrating cell. The Ca^2+^ gradient is created by the differential distribution of plasma membrane Ca^2+^-ATPase (PMCA, shown as P in the illustration), resulting in higher pump activity to move cytosolic Ca^2+^ out of the cell in the front than the back. Low Ca^2+^ in the front “starves” myosin light chain kinase (MLCK), which is essential for its reactivity to local Ca^2+^ pulses. High Ca^2+^ in the back facilitates the turnover of stable focal adhesion complexes. (See Figure 4 and the text for more details.)

**Figure 3:**
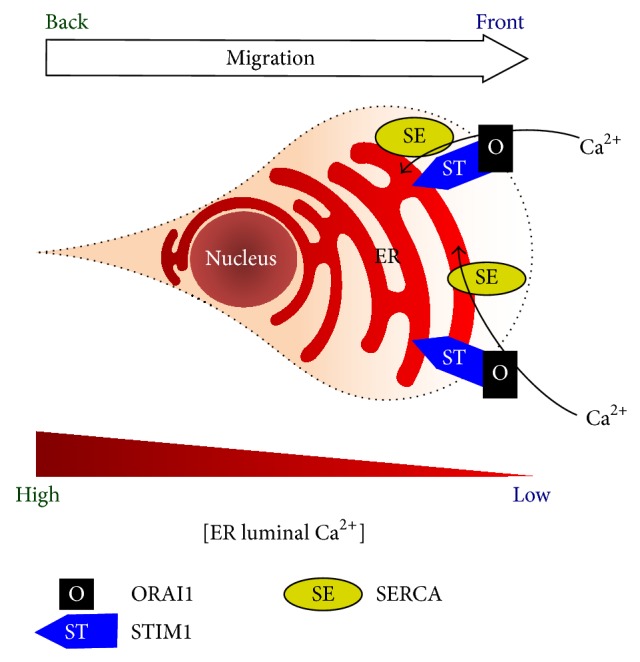
Internal Ca^2+^ storage is maintained through the differential activities of store-operated Ca^2+^ (SOC) influx during cell migration. Repetitive Ca^2+^ release from inositol triphosphate (IP_3_) receptors causes the depletion of Ca^2+^ in the endoplasmic reticulum (ER) near the leading edge. Such Ca^2+^ depletion activates STIM1 (shown as ST in the illustration), which is translocated to the ER-plasma membrane junction to open Ca^2+^ channels ORAI1 (shown as O in the illustration). The inward Ca^2+^ current through ORAI1 will further travel into the ER via sarcoplasmic/endoplasmic reticular Ca^2+^-ATPase (SERCA, shown as SE in the illustration). In migrating cells, STIM1 proteins are enriched in the front ER to maintain Ca^2+^ homeostasis, which is essential for proper polarity and motility.

**Figure 4:**
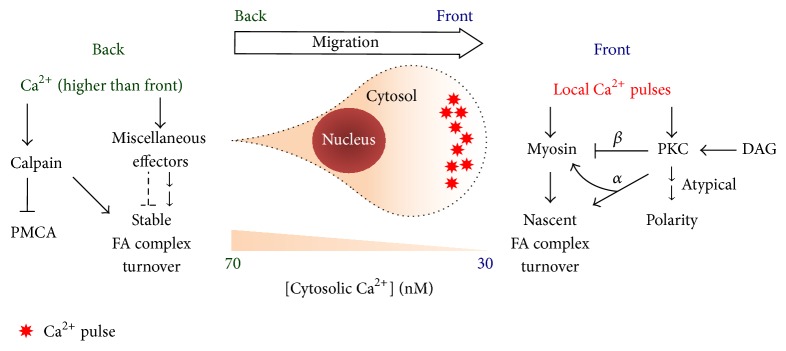
Ca^2+^ is temporally and spatially regulated to control the cell migration machinery via a wide variety of effectors. (Right) In the front, Ca^2+^ activates myosin and protein kinase C (PKC) for the maintenance of polarity and establishment of nascent cell-matrix adhesion. (Left) In the back, Ca^2+^ mediates calpain and miscellaneous focal-adhesion (FA) regulators, so proper disassembly of stable FA complexes can proceed. DAG: diacylglycerol; PMCA: plasma membrane Ca^2+^-ATPase.

**Table 1:** Roles of store-operated Ca^2+^ (SOC) influx on cancer cell migration.

Gene(s)/Protein(s)	Cell type	Highlight	Target(s)	Reference
ORAI1	Esophageal squamous cell carcinoma (ESCC)	ORAI1 controls intracellular Ca^2+^ oscillations	N.A.	[105]

ORAI1 and STIM1	Clear cell renal cell carcinoma (ccRCC)	ORAI1 and STIM1 regulate cell proliferation and migration	N.A.	[106]

ORAI1 and STIM2	Melanoma cell lines	ORAI1 and STIM2 control melanoma growth and invasion in opposite manners	N.A.	[107]

ORAI1	Breast cancer cells	cAMP-PKA pathway decreases SK3 channel and SK3-ORAI1 complex activities, reducing Ca^2+^ entry and cancer cell migration	cAMP, PKA	[108]

STIM1	Breast cancer cell line MDA-MB-435s	Targeting SK3-ORAI1 in lipid rafts may inhibit bone metastasis	SK3	[109]

STIM1	Cervical cancer cell lines (SiHa, HT-3, CaSki, and HeLa)	HDAC6 may disrupt STIM1-mediated SOC influx and block malignant cell behavior	HDAC6	[110]

ORAI1 and STIM1	Glioblastoma multiforme (GBM)	STIM1 and ORAI1 affect the invasion of GBM cells	N.A.	[111]

ORAI1	Human T cell leukemia line, Jurkat cell	Monoclonal antibodies against ORAI1 reduce SOC influx, NFAT transcription, and cytokine release	N.A.	[112]

ORAI1	Human prostate cancer (PCa) cell	Bisphenol A pretreatment enhances SOC influx and ORAI1 protein in LNCaP cells; it also induces PCa cells migration	N.A.	[113]

STIM1	Cervical cancer cell	STIM1 regulates actomyosin reorganization and contractile forces to control cell migration	Actomyosin	[114]

STIM1	Hepatocellular carcinoma and hepatocyte cell lines	STIM1 level predicts prognosis in patients of liver cancer	N.A.	[115]

STIM1	Human epidermoid carcinoma A431 cells	STIM1 regulates SOC influx, cell proliferation, and tumorigenicity	N.A.	[116]

STIM1	Cervical cancer SiHa and CaSki cell lines	STIM1 regulates cervical cancer growth, migration, and angiogenesis	Focal adhesion, Pyk2	[7]

ORAI1 and STIM1	MDA-MB-231 human breast cancer cells	Blocking STIM1 or ORAI1 using RNA interference or small molecule inhibitors decreased tumor metastasis in animal models	Focal adhesion	[82]

**Table 2:** Roles of store-operated SOC influx on the motility of nonmalignant cells.

Gene(s)/Protein(s)	Cell type	Highlight	Target(s)	Reference
STIM1	Endothelial progenitor cells (EPCs)	STIM1 affects EPCs proliferation and migration after vascular injury by regulating Ca^2+^ levels	N.A.	[117]

ORAI1	HEK293	Selective activation of NFAT by ORAI1	NFAT	[118]

STIM1	Endothelial leader cells	Cells employ an integrated and polarized Ca^2+^ signalling system for directed cell migration	PLC pathway	[25]

ORAI1	Keratinocytes	ORAI1-mediated Ca^2+^ entry enhances the turnover of focal adhesion through PKC*β*, calpain, and focal adhesion kinase	PKC pathway	[119]

ORAI1 and STIM1	Retinal pigment epithelial cells (ARPE-19 cell line)	STIM1, ORAI1, ERK 1/2, and Akt determine EGF-mediated cell growth	MAPK pathway	[120]

STIM1	HEK293	STIM regulates focal adhesion dynamics	Focal adhesion	[121]

ORAI1 and STIM1	Airway smooth muscle cell (ASMC)	STIM1 or ORAI1 controls PDGF-mediated ASMC proliferation and chemotactic migration	N.A.	[122]

ORAI1 and STIM1	ASMC	STIM1 and ORAI1 control PDGF-induced cell migration and Ca^2+^ influx	N.A.	[123]

STIM1	Intestinal epithelial cell (IEC)	Polyamines control TRPC1-mediated Ca^2+^ signaling and cell migration via differential STIM1 and STIM2 levels	TRPC1	[124]

ORAI1 and STIM1	Vascular smooth muscle cells (VSMC)	STIM1- and ORAI1-mediated SOC influx regulates angiotensin II-induced VSMC proliferation	N.A.	[125]

STIM1	EPCs	STIM1 regulates the proliferation and migration of EPCs	N.A.	[126]

ORAI1 and STIM1	VSMC	STIM1 and ORAI1 regulate PDGF-mediated Ca^2+^ entry and migration in VSMC	N.A.	[127]

ORAI1 and STIM1	VSMC	Knockdown of STIM1 and ORAI1, but not STIM2, Orai2, or Orai3, reduces VSMC proliferation and migration	N.A.	[128]
